# Cardiovascular determinants of prognosis in normotensive hemodialysis patients

**DOI:** 10.1186/1471-2369-13-115

**Published:** 2012-09-20

**Authors:** Wen-Chung Yu, Yao-Ping Lin, Shao-Yuan Chuang, I-Feng Lin, Chen-Huan Chenb

**Affiliations:** 1 Department of Medicine, Taipei, Taiwan; 2 Department of Medical Research and Education, Taipei Veterans General Hospital, Taipei, Taiwan; 3 Institute of Population Health Sciences, National Health Research Institutes, Taipei, Taiwan; 4 Department of Public Health, Taipei, Taiwan; 5 Department of Medicine,, National Yang-Ming University, Taipei, Taiwan

**Keywords:** Arterial stiffness, End-stage renal disease, Hypertension, Left ventricular function, Mortality

## Abstract

**Background:**

Normotension has been hold to be the goal of hemodialysis. It remains obscure which cardiovascular parameter determines the prognosis in these normotensive hemodialysis patients.

**Methods:**

We prospectively enrolled 145 hemodialysis patients, who had attained normotension without anti-hypertensive medications, and followed them for 72.6 ± 28.5 months. Important cardiovascular parameters were obtained at enrollment. Predictors for all-cause and cardiovascular mortalities were identified with the Cox model.

**Results:**

There were 45 (18 cardiovascular/27 non-cardiovascular) deaths occurred during follow-up. Age, diabetes, left ventricular mass index (LVMI), left ventricular ejection fraction (LVEF), carotid intima-media thickness (CIMT), and aortic pulse wave velocity (PWV) were significant predictors for all-cause and cardiovascular mortalities. After adjustment for age and diabetes, only LVEF was significantly associated with all-cause mortality. LVEF was significantly associated with cardiovascular mortality. LVEF remained as a significant independent predictor of cardiovascular death after adjusting for age, diabetes, LVMI, CIMT, or PWV, respectively.

**Conclusion:**

LVEF is the independent predictor for all-cause and cardiovascular mortalities in the normotensive hemodialysis patients.

## Background

There is a globally rising prevalence of end stage renal disease (ESRD) patients receiving regular hemodialysis. Despite advances in dialysis facilities and techniques, the mortality remains high [1]. Hypertension and hemodynamic overload are widely accepted as the main culprits of the cardiovascular structural and functional alterations and the cardiovascular disease, the leading cause of death in these patients [2]. Under hypertension and hemodynamic overload, the cardiac factors, including left ventricular hypertrophy [3] and left ventricular dysfunction, [4] have been proposed as the major cardiac determinants of cardiovascular mortality in the hemodialysis patients. In addition, arterial factors, including arterial stiffness, [5] arterial wave reflections, [6] carotid intima-media thickness (CIMT), [7] and carotid incremental elastic modulus (Einc) [8] have also been recognized as predictors of cardiovascular outcomes.

Lowering blood pressure (BP) has been widely accepted to reduce all-cause and cardiovascular mortalities in patients on dialysis [9]. Most investigators agree that optimization of fluid status is the initial intervention for most hypertensive hemodialysis patients. Patients are instructed to lead a disciplined life with decreasing salt intake, maintaining inter-dialytic weight gain within narrow ranges, while simultaneously keeping adequate calorie intake. Data from the Tassin experience [10] and the DRIP trial [11] suggest that reducing dry weight does improve BP in a significant number of patients. However, the conundrum is that what cardiac or arterial factors govern mortality when the hemodialysis patients overcome all the obstacles to attain normotension or dry weight?

In this study, we enrolled hemodialysis patients who had already attained dry weight, i.e., the body weight at the end of dialysis at which the patient could remain normotensive until the next dialysis session without the need for antihypertensive agents despite interdialytic weight gain [10]. All patients received baseline comprehensive cardiac and arterial structure and function assessments. We aimed to investigate the cardiovascular determinants of all-cause and cardiovascular mortalities, and the potential therapeutic targets in this unique population.

## Methods

### Study Patients

The dialysis population consisted of 812 patients treated at our institution, a nearby community hospital and three dialysis centers during the period of January 1998 through December 2002. Patients were considered eligible for inclusion when (1) they had been on maintenance hemodialysis for at least 3 months, and (2) their systolic blood pressure (SBP) was below 140 mmHg and diastolic blood pressure (DBP) below 90 mmHg without necessity of anti-hypertensive medications for at least 90% of 25 consecutive pre-dialysis measurements. There were 620 patients excluded due to hypertension, 38 patients due to acute diseases and 9 patients due to refusal.

We consecutively enrolled 145 hemodialysis patients (79 men, 66 women) with a mean age of 55.0 ± 15.0 years. Sixteen patients (11%) were diabetics. Each subject provided written informed consent, which was approved by our institutional review board of Taipei Veterans General Hospital. All participants received a comprehensive cardiovascular examination and blood tests on the mid-week non-dialysis day [8].

### Hemodialysis procedures

The patients received 4-hour dialysis session thrice-weekly using 1.6 m^2^ surface area dialyzers with bicarbonate-based dialysate (Na^+^ 140 meq/liter, HCO_3_^-^ 39 meq/liter, K^+^ 2.0 meq/liter, Ca^2+^ 3.0 meq/liter and Mg^2+^ 1.0 meq/liter). In addition to baseline echocardiographic and vascular examinations**,** they were assessed via meticulous physical examinations and chest X-ray and/or echocardiography for adjustment of fluid status during the follow-up period. Dry weight was defined as that proposed by Charra et al. [10]. If symptoms and signs of fluid overload were noted, the exceeded volume was ultrafiltrated during the dialysis session or via additional sessions. All patients received subcutaneous recombinant erythropoietin at a mean dosage of 20,000U per month to keep their levels of hematocrit up to 30% according to our National Health Insurance guideline.

### Arterial factors

Arterial structure and function were assessed using ultrasound (SONOS 5500, Philips, Andover, MA, USA) and arterial tonometry. Supine brachial SBP and DBP were the average of 4 measurements with an oscillometric device. Pulse pressure (PP) was the difference between SBP and DBP. Mean blood pressure (MBP) was DBP + 1/3 PP.

The measured arterial structural parameter was CIMT [12]. The arterial functional parameters included aortic pulse wave velocity (PWV), carotid augmentation index, and carotid E_inc_, which were acquired as previously reported [13]. The increased CIMT was defined as CIMT > 0.9 mm and the increased aortic PWV as PWV > 12 m/s [14].

### Cardiac factors

Left ventricular mass and ejection fraction were calculated from M-mode measurements according to the recommendation of the American Association of Echocardiography [15]. Left ventricular mass index (LVMI) was left ventricular mass divided by height^2.7^[16]. LVH was defined as LVMI >50 g/height^2.7^ for male and >47 g/height^2.7^ for female.

Supine BP immediately before each dialysis session was measured using an oscillometric BP monitor. Values from 25 consecutive dialysis sessions before the cardiovascular examination were averaged as pre-dialysis BP.

### Uremia related modulators and laboratory evaluation

The clinical biochemistry data was obtained by the averages of two-month data preceding patients’ entry into this study. The adequacy of hemodialysis dosage was indexed by the formulae Kt/V.

Hemoglobin and hematocrit were measured by CELL-DYNE 1400 (Abbot Laboratories, Abbott park, IL, U.S.A.).

### Follow-up

The date and causes of death for the deceased patients were collected by both telephone contact and review of hospital charts and death certificates coded according to the International Classification of Disease, Ninth Revision (ICD-9). The ICD-9 codes used for cardiovascular death were 390–459.

### Statistical analysis

Data were expressed as mean ± SD for continuous variables and as proportions for categorical variables. Between-group comparisons were performed by one-way ANOVA with Duncan post-hoc test for continuous variables and by Chi-square test for categorical variables. Predictors for all-cause and cardiovascular mortalities were identified with the Cox proportional hazard regression. Survival curves were plotted using the Kaplan-Meier method and assessed using the log-rank test. Values of *P* <0.05 was considered as statistically significant. All statistic procedures were carried out using SPSS, version 17.0.

## Results

The characteristics of the study patients are shown in Table 1. The previous cardiovascular diseases including 3 strokes, 2 coronary artery diseases and 14 hypertensive cardiovascular diseases. There were no significant difference of the types of previous cardiovascular disease among the survivors, non-cardiovascular mortality, and cardiovascular mortality. During a mean follow-up of 72.6 ± 28.5 months, no patients received renal transplant or lost to follow up. There were 18 cardiovascular and 27 non-cardiovascular deaths. The mean annual all-cause mortality was 5.1% and cardiovascular mortality was 2.1%. There were 9 patients with abnormal LVEF, 77 patients with LVH, 39 patients with increased thickness of CIMT, and 21 patients with increased PWV. Patients died from cardiovascular causes were older than the survivors and were more likely to have type 2 diabetes mellitus. They also had significantly higher LVMI, lower LVEF, greater CIMT, and faster PWV. In comparison with those who died from non-cardiovascular causes, patients died from cardiovascular causes also had significantly higher LVMI, lower LVEF, greater CIMT, and faster PWV.

**Table 1:** Baseline characteristics of study patients

**Variables**	**Survivors (n = 100)**	**Non-cardiovascular death (n = 27)**	**Cardiovascular death (n = 18)**
Age (year)	48.8 ± 13.0*	67.3 ± 13.7	69.1 ± 8.3
Male gender,%	53%	61%	55%
Weight (kg)	59.3 ± 10.5	60.4 ± 8.2	59.7 ± 9.7
Height (cm)	161 ± 9	161 ± 7	158 ± 9
BMI (kg/m^2^)	22.8 ± 3.3	23.3 ± 3.0	23.7 ± 2.8
Diabetes (%)	4%*	18%^#^	41%
History of cardiovascular disease (%)	6%*	29%	31%
Hypertension history (%)	17.0	22.2	27.1
Previous hypertension duration (years)	13.3 ± 4.7	12.6 ± 3.2	13.8 ± 5.0
Medications			
Vitamin D receptor agonists (%)	15.1	22.0	18
Lipid lowering agents (%)	28.3	22.2	20
Dialysis duration (days)	2178 ± 2006	978 ± 1018	1508 ± 1124
Systolic BP (mmHg)	114 ± 17	107 ± 19	116 ± 15
Diastolic BP (mmHg)	66 ± 12	61 ± 10	68 ± 10
Pulse BP (mmHg)	47 ± 9	46 ± 13	49 ± 11
Systolic BPavg (mmHg)	126 ± 19	119 ± 19	131 ± 18
Diastolic BPavg (mmHg)	75 ± 10	70 ± 9	76 ± 8
Pulse BPavg (mmHg)	51 ± 10	49 ± 13^#^	55 ± 13
Hematocrit (%)	31.0 ± 4.2*	30.0 ± 2.9	29.3 ± 2.7
Hemoglobin (g/L)	103 ± 15	100 ± 11	100 ± 8
Kt/V	1.56 ± 0.31	1.50 ± 0.18	1.44 ± 0.22
LVMI (g/height^2.7^)	50.3 ± 14.1*	51.6 ± 15.3^#^	72.1 ±31.0
LV EF (%)	71 ± 8*	72 ± 13^#^	62 ±17
Carotid IMT (μm)	774 ± 166*	875 ± 146^#^	1015 ± 249
Carotid Augmentation Index (%)	13.8 ± 20.3	14.5 ± 14.5	19.3 ± 20.6
Carotid E_inc_ (kPa*10^3^)	0.39 ± 0.20	0.41 ± 0.26	0.45 ± 0.17
PWV (m/s)	8.3 ± 3.0*	10.3 ± 4.2^#^	12.8 ± 2.2

*: P value <0.05 for comparison between survivors and cardiovascular deaths.

#: P value < 0.05 for comparison between non-cardiovascular and cardiovascular deaths. BMI = body mass index; BP = blood pressure; BPavg = pre-dialysis blood pressure averaged from 25 consecutive hemodialysis sessions; Einc = incremental elastic modulus; IMT = intima-media thickness; LV EF = left ventricular ejection fraction; LVMI = left ventricular mass index; PWV = pulse wave velocity.

Univariate analysis of significant predictors for all-cause mortality included age, diabetes mellitus, pre-dialysis DBP, history of cardiovascular disease, hematocrit, LVMI, LVEF, CIMT, and PWV (Table 2). Regarding cardiovascular mortality, age, diabetes mellitus, history of cardiovascular disease, LVMI, LVEF, CIMT, and PWV were significant predictors (Table 2).

**Table 2:** Hazard ratios of all-cause and cardiovascular mortality based on univariate Cox regression models

**Variable**	**All-cause mortality**		**Cardiovascular mortality**	
	**Hazard Ratio (95% CI)**	***P*value**	**Hazard Ratio (95% CI)**	***P*value**
Age (year)	1.08 (1.05-1.11)	<0.001	1.09 (1.04-1.14)	<0.001
Sex (men = 1, female = 0)	1.15 (0.64-2.03)	0.6	0.91 (0.35-2.38)	0.9
BMI (kg/m^2^)	1.02 (0.94-1.11)	0.7	1.04 (0.91-1.20)	0.5
Diabetes (yes =1, no = 0)	4.47 (2.28-8.76)	<0.001	7.80 (2.91-20.92)	<0.001
History of cardiovascular disease (yes = 1, no = 0)	2.85 (1.47-5.45)	0.002	3.10 (1.08-8.93)	0.04
Dialysis duration (days)	1.0 (0.99-1.00)	0.4	1.0 (1.00-1.00)	0.4
Systolic BP (mmHg)	0.99 (0.98-1.01)	0.9	1.01 (0.98-1.04)	0.4
Diastolic BP (mmHg)	0.98 (0.96-1.004)	0.9	1.02 (0.97-1.06)	0.7
Pulse BP (mmHg)	1.00 (0.97-1.03)	0.9	1.03 (0.99-1.08)	0.5
Systolic BPavg (mmHg)	0.99 (0.98-1.006)	0.2	1.01 (0.99-1.04)	0.3
Diastolic BPavg (mmHg)	0.97 (0.94-0.99)	0.03	1.00 (0.93-1.07)	0.9
Pulse BPavg (mmHg)	1.00 (0.98-1.02)	0.7	1.03 (0.99-1.06)	0.2
Hematocrit (%)	0.91 (0.84-0.99)	0.04	0.91 (0.78-1.04)	0.2
Kt/V	0.43 (0.16-1.15)	0.09	0.52 (0.11-2.49)	0.4
LVMI (g/height^2.7^)	1.02 (1.005-1.04)	0.01	1.04 (1.02-1.06)	<0.001
LV EF (%)	0.97 (0.947-0.998)	0.03	0.94 (0.91-0.97)	<0.001
Aorta dimension (mm)	1.69 (0.73-3.92)	0.2	1.36 (0.34-5.45)	0.7
Carotid IMT(μm)	1.002 (1.001-1.004)	0.02	1.0053 (1.001-1.006)	0.003
Carotid augmentation index (%)	1.00 (0.98-1.02)	0.9	1.01 (0.98-1.04)	0.4
Carotid Einc (kPa *10^3^)	2.45 (0.70-8.56)	0.2	2.61 (0.35-19.34)	0.4
Aortic PWV (m/s)	1.13 (1.06-1.20)	<0.001	1.19 (1.09-1.29)	<0.001

BMI = body mass index; BP = blood pressure; BPavg = pre-dialysis blood pressure averaged from 25 consecutive hemodialysis sessions; Einc = incremental elastic modulus; IMT = intima-media thickness; LV EF = left ventricular ejection fraction; LVMI = left ventricular mass index; PWV = pulse wave velocity.

Among the four key cardiac (LVMI, LVEF) and arterial factors (CIMT, PWV) that could have prognostic impacts, only CIMT (r = 0.46, P <0.001) and PWV (r = 0.59, P <0.001) were significantly correlated with age.

For all-cause mortality (Table 3), only LVEF was an independent predictor, when age and diabetes mellitus were accounted for (Table 3, Model 1). CIMT and PWV were not significant independent predictors for all-cause mortality after adjusted for age and diabetes (Tables 3, Model 2). LVEF remained as a significant predictor when CIMT was entered into the model (Table 3, Model 3). LVEF also remained as a significant predictor when aortic PWV was entered into the model (Table 3, Model 4).

**Table 3:** Hazard ratios of all-cause mortality based on multi-variate Cox regression models

	**Model 1**		**Model 2**		**Model 3**		**Model 4**	
	**Hazard Ratio (95% CI)**	***P*value**	**Hazard Ratio (95% CI)**	***P*value**	**Hazard Ratio (95% CI)**	***P*value**	**Hazard Ratio (95% CI)**	***P*value**
Age (year)	1.07 (1.04-1.10)	<0.001	1.07 (1.04-1.11)	<0.001	1.07 (1.04-1.10)	<0.001	1.07 (1.05-1.11	<0.001
Diabetes (yes = 1, no = 0)	1.81 (0.89-3.70)	0.1	1.93 (0.86-4.2)	0.1	1.86 (0.92-3.76)	0.08	2.14 (0.96-4.76)	0.06
LVMI (g/height^2.7^)	1.003 (0.99-1.02)	0.5						
LV EF (%)	0.97 (0.95-0.99)	0.015			0.97 (0.95-0.99)	0.008	0.97 (0.95-0.99)	0.01
Carotid IMT (μm)			1.05 (0.87-1.26)	0.6	1.07 (0.89-1.29)	0.4		
Aortic PWV (m/s)			0.96 (0.87-1.06)	0.5			0.96 (0.88-1.06)	0.4

For cardiovascular mortality, LVEF was also an independent predictor. We used restricted models for multi-variate analysis because of the low number of cardiovascular death. LVEF remained as a significant predictor of cardiovascular death in analyses adjusting for age, diabetes, LVMI, CIMT, or PWV, respectively (Table 4).

**Table 4:** Hazard ratios of cardiovascular mortality based on restricted multi-variate Cox regression models

	**LVEF (%)**		
**Adjusted variable**	**Hazard ratio**	**95% CI**	**P value**
Age (year)	0.95	0.92 - 0.98	0.001
Diabetes (yes = 1, no = 0)	0.95	0.92 - 0.98	0.004
LVMI (g/height^2.7^)	0.96	0.93 - 0.99	0.03
Carotid IMT (μm)	0.94	0.91 - 0.98	0.001
Aortic PWV (m/s)	0.95	0.92 - 0.99	0.006

**Figure 1:**
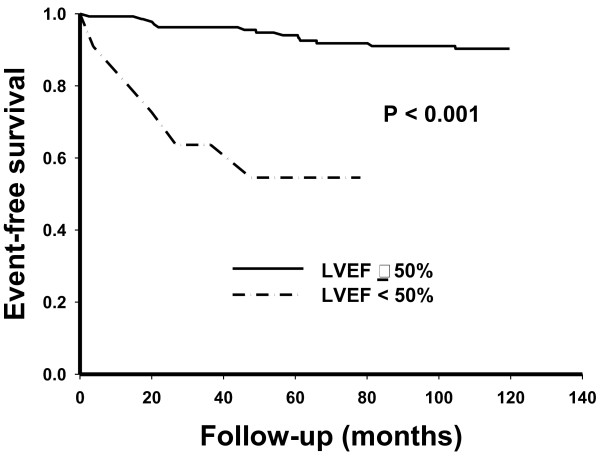
**Cardiovascular (CV) survival curves for left ventricular ejection fraction (LVEF) (Panel A, log rank test *P *< 0.001) and left ventricular mass index (LVMI) (Panel B, log rank test *P *= 0.03).**

In the normotensive hemodialysis patients, LVEF <50% (median value) predicted cardiovascular mortality (Figure 1). We compared blood pressure, LVMI, carotid IMT, carotid augmentation index and PWV between groups of above and below LVEF 50%. There was no significant difference of blood pressure (systolic BP:113 ± 18 mmHg vs. 108 ± 14 mmHg, p = 0.3; diastolic BP: 66 ± 12 mmHg vs. 67 ± 7 mmHg, p = 0.7; pulse pressue: 47 ± 11 vs. 43 ± 7 mmHg, p = 0.3), carotid IMT (813 ± 178 μm vs. 899 ± 263 μm, p = 0.1 ), carotid augmentation index (14.7 ± 19.3 m vs. 14.6 ± 17.9 m, p = 0.9) and PWV (9.0 ± 3.5 m/s vs. 9.9 ± 2.9 m/s, p = 0.4) between groups of above and below LVEF 50% ; only borderline significant for LVMI (51 ± 15 gm vs. 72 ± 38, p = 0.09).

## Discussion

The present study demonstrated that even in hemodialysis patients who had already attained normotension/dry weight, reduced LVEF was an independent predictor for both all-cause and cardiovascular mortalities. Compared with the general hemodialysis patients, [17] we also confirmed that the normotensive hemodialysis patients had a relatively favorable prognosis.

Reduced LVEF is associated with adverse cardiovascular events in ESRD patients [18]. It should be noted that that even mild LV systolic dysfunction may significantly deteriorate the prognosis in the general hemodialysis patients [19]. In a 7 years followed up of 1,254 incident hemodialysis patients with 71.9% being hypertensive, reduced LVEF on starting hemodialysis was a strong independent predictor for all-cause and cardiovascular deaths [19].

High BP has well been recognized as a major determinant of deranged cardiovascular structure and function and an independent predictor of cardiovascular risk in the general population and in patients with ESRD [13]. Therefore, lowering BP by adjusting dry-weight and adding necessary antihypertensive drugs has been proposed to diminish the cardiovascular mal-adaptation. In a previous cross-sectional study, we have observed favorable cardiovascular structural and functional remodeling in the normotensive hemodialysis patients when compared with the hypertensive hemodialysis counterparts [12]. However, attaining normotension could not fully reverse the cardiovascular structure and function to normal [12]. These patients still have increased LVMI due to persistent flow overload and subclinical left ventricular dysfunction. The present prospective study further highlights the importance of the persistent left ventricular structural and functional parameters in determining prognosis.

We have shown previously that volume status was closely related to the structure and function of the large artery in hemodialysis patients [20]. Therefore, the arterial structure (aortic diameter and CIMT) and function (PWV, carotid augmentation index, and carotid Einc) could be modified by probing dry weight to reduce BP. We reasoned that the arterial factors might have been improved substantially in normotensive state, and thus might become less important in predicting outcomes. The present study supports the reasoning and also implies that targeted interventions towards cardiac structure and function may further improve clinical outcomes in the normotensive hemodialysis patients.

The uremic milieu other than the pressure/volume overload, such as anemia, deranged calcium-phosphate homeostasis, secondary hyperparathyroidism, sympathetic overactivity, increased oxidative stress, chronic inflammation and accumulated uremic toxins, etc. already exist in the predialysis stages. All these factors intricately participate in the pathogenesis of cardiovascular derangements, [21] and the cardiovascular changes are already quite pronounced by the time patients commence hemodialysis. Therapy targeted toward single factor usually fails to improve hemodialysis prognosis. For example, a 12-month treatment with ramipril did not cause significant regression of left ventricular hypertrophy in normotensive hemodialysis patients [22]. Therefore, a multi-disciplined interventional strategy may be required to offer optimal cardiac and overall outcomes.

Notwithstanding better BP control, demographic parameters such as age and diabetes mellitus still played pivotal roles in affecting the prognosis of our normotensive hemodialysis patients. Actually, patients’ age per se exerts impacts on cardiovascular structure and function [23]. Similar to the general hemodialysis patients, diabetes also exerted deleterious effects on clinical outcomes in our normotensive hemodialysis patients. Diabetes is a well known risk factor for ischemic heart disease, reduced cardiac function, and cardiac events in uremic patients [24]. Given the increasing prevalence of elderly and diabetic dialysis patients, [25] individualized treatment plans designed for patients with different demographic features may be needed, even if they have already achieved normotension by hemodialysis.

The present study is limited by the small number of enrolled patients as relatively few HD patients can remain normotensive without taking antihypertensives. Further multicenter studies with longer prospective follow-up for more events would facilitate detailed multivariate analysis by taking into account more cardiovascular risk factors as the nutrition status, inflammatory markers, [26] and calcium-phosphate homeostasis, etc.

## Conclusions

In conclusion, the normotensive hemodialysis patients usually have relatively good all-cause and cardiovascular mortality outcomes. LVEF appears to be a major predictor for all-cause and cardiovascular mortalities in this unique population. Future studies are required to investigate whether targeted interventions to improve cardiac systolic function may further improve clinical outcomes in these patients.

## Competing interests

The authors declare that they have no competing interests.

## Authors' contributions

W-CY: Design, performed cardiovascular examination, drafting and revising the manuscript. Y-PL: Design, data collection, recruitment, drafting and revising the manuscript. S-YC: Conceived the study, participated in statistical analysis and revising the manuscript. I-FL: Conceived the study, participated in statistical analysis and revising the manuscript. C-HC: Conceived the study, acquired funding, provided overarching supervision, manuscript revision. All authors have read and approved the final manuscript.

## Pre-publication history

The pre-publication history for this paper can be accessed here:

http://www.biomedcentral.com/1471-2369/13/115/prepub
